# Economic potential of abbreviated breast MRI for screening women with dense breast tissue for breast cancer

**DOI:** 10.1007/s00330-022-08777-5

**Published:** 2022-04-28

**Authors:** Fabian Tollens, Pascal A. T. Baltzer, Matthias Dietzel, Moritz L. Schnitzer, Vincent Schwarze, Wolfgang G. Kunz, Johann Rink, Johannes Rübenthaler, Matthias F. Froelich, Stefan O. Schönberg, Clemens G. Kaiser

**Affiliations:** 1 Department of Radiology and Nuclear Medicine, University Medical Centre Mannheim, Medical Faculty Mannheim-University of Heidelberg, Theodor-Kutzer-Ufer 1-3, D-68167 Mannheim, Germany; 2 Department of Biomedical Imaging and Image-Guided Therapy, Vienna General Hospital, Medical University of Vienna, Währinger Gürtel 18-20, 1090 Vienna, Austria; 3 Department of Radiology, Friedrich-Alexander-University Hospital Erlangen, Maximiliansplatz 1, D-91054 Erlangen, Germany; 4 Department of Radiology, University Hospital, LMU Munich, Marchioninistr. 15, D-81377 Munich, Germany

**Keywords:** Mammography, Magnetic resonance imaging, Breast neoplasms, Cost-effectiveness analysis, Screening

## Abstract

**Objectives:**

Abbreviated breast MRI (AB-MRI) was introduced to reduce both examination and image reading times and to improve cost-effectiveness of breast cancer screening. The aim of this model-based economic study was to analyze the cost-effectiveness of full protocol breast MRI (FB-MRI) vs. AB-MRI in screening women with dense breast tissue for breast cancer.

**Methods:**

Decision analysis and a Markov model were designed to model the cumulative costs and effects of biennial screening in terms of quality-adjusted life years (QALYs) from a US healthcare system perspective. Model input parameters for a cohort of women with dense breast tissue were adopted from recent literature. The impact of varying AB-MRI costs per examination as well as specificity on the resulting cost-effectiveness was modeled within deterministic sensitivity analyses.

**Results:**

At an assumed cost per examination of $ 263 for AB-MRI (84% of the cost of a FB-MRI examination), the discounted cumulative costs of both MR-based strategies accounted comparably. Reducing the costs of AB-MRI below $ 259 (82% of the cost of a FB-MRI examination, respectively), the incremental cost-effectiveness ratio of FB-MRI exceeded the willingness to pay threshold and the AB-MRI-strategy should be considered preferable in terms of cost-effectiveness.

**Conclusions:**

Our preliminary findings indicate that AB-MRI may be considered cost-effective compared to FB-MRI for screening women with dense breast tissue for breast cancer, as long as the costs per examination do not exceed 82% of the cost of a FB-MRI examination.

**Key Points:**

• *Cost-effectiveness of abbreviated breast MRI is affected by reductions in specificity and resulting false positive findings and increased recall rates.*

• *Abbreviated breast MRI may be cost-effective up to a cost per examination of 82% of the cost of a full protocol examination.*

• *Abbreviated breast MRI could be an economically preferable alternative to full protocol breast MRI in screening women with dense breast tissue.*

## Introduction

The superior accuracy of breast MRI in comparison with conventional imaging techniques in screening women at high risk for breast cancer has been suggested by a number of studies; several recent prospective multi-center trials have compared breast MRI to conventional imaging techniques, such as conventional mammography (XM) and ultrasound (US), underlining a higher sensitivity at comparable specificity levels [1–3]. However, current international guidelines still do not recommend breast MRI as a screening tool outside of high-risk screening collectives [4–6].

Apart from high-risk collectives, the superior diagnostic performance has also been confirmed in screening women at intermediate risk of breast cancer due to their elevated breast density [7, 8]. Dense breast tissue is associated with an increased risk of breast cancer along with a decreased sensitivity in conventional breast imaging (ACR BI-RADS categories C or D) [9].

In order to cope with the scarcity in resource allocation and to make breast MRI more affordable, abbreviated protocols (AB-MRI) have been introduced offering similar sensitivity in comparison to full protocol breast MRI (FB-MRI), yet at the expense of decreased specificity. Initially, AB-MRI was defined as solely pre- and post-contrast sequences with subtracted and maximum-intensity projection images [10]. Recently, a variety of abbreviated protocols have been evaluated. These included additional T2-weighted images, ultrafast contrast-enhanced sequences, or diffusion-weighted images as a contrast-free alternative — all reporting varying specificities [11–14]**.**

The prospective trials of Comstock and Bakker et al have reported similar sensitivities of AB-MRI and FB-MRI in women of intermediate risk. Comstock et al described a specificity of AB-MRI of 87%, whereas Bakker et al found a specificity of 92% for the full protocol, in line with prior findings [10].

The lower specificity of AB-MRI is associated with a higher number of false positive findings and increased recall rates. Both techniques have been described to be cost-effective in screening women at intermediate risk with an incremental cost-effectiveness ratio (ICER) below commonly accepted willingness to pay thresholds of $100,000 per quality-adjusted life year (QALY) gained [15–17].

The lack of a broadly accepted definition for both techniques — abbreviated as well as full-scale protocols — makes the evaluation of AB-MRI in general difficult as every approach leads to a different diagnostic accuracy.

Based on recent data, reductions in specificity that are associated with abbreviating breast MRI protocols were assumed as the key determinant to model the differences between full-scale and abbreviated protocols in the present study.

Consecutively, we aim to investigate the role of AB-MRI in screening women with dense breast tissue in relation to FB-MRI with a focus on the following objectives:

1. To evaluate the economic potential of AB-MRI compared to FB-MRI,
2. To examine various constellations of diagnostic performance parameters and costs with regard to cost-effectiveness, and
3. To determine a “cut-off” value that would allow AB-MRI to be cost-effective.

## Material and methods

### Screening collective and input parameters

This cost-effectiveness analysis is based on recently published data on AB-MRI and FB-MRI, evaluated in prospective, multi-center screening trials and compared to x-ray-based techniques, i.e., x-ray mammography (XM) and digital breast tomosynthesis (DBT) [7, 8]. The underlying studies revealed unprecedented data on screening women at intermediate risk of breast cancer due to dense breast tissue. Both average age and pre-test probability of a malignant lesion were comparable in the screening collectives, which were reported at about 55 years and 1.6%, respectively. While the sensitivity of AB-MRI and FB-MRI was equivalent at 95.7% and 95.2%, respectively, the specificity of AB-MRI was significantly lower compared to a full protocol examination (86.7% and 92.0%, respectively). Diagnostic performance of XM was estimated based on recent literature [2, 3, 18–20].

#### Input parameters

Input parameters for this economic evaluation were collected from recent literature (Table 1) closely following international recommendations on the conduct and methodological practice of cost-effectiveness analyses [22, 33, 34].

**Table 1:** Model input parameters. Model input parameters for the decision analysis and Markov modelling that have been published recently and refined for this analysis and patient collective [15, 17]

Variable	Estimation	Source
Pre-test probability of malignant lesion	1.62%	[7, 8]
Average age at screening	55	[7, 8]
Screening interval	2 years	
Incidence of breast cancer	0.40%	[21]
Assumed WTP	$ 100,000	[22]
Discount rate	3.00%	[22]
Diagnostic test performances		
Sensitivity of XM	41.2%	[2, 19, 20]
Specificity of XM	90.0%	[18]
Sensitivity of DBT	39.1%	[7]
Specificity of DBT	97.4%	[7]
Sensitivity of AB-MRI	95.7%	[7]
Specificity of AB-MRI	86.7%	[7]
Sensitivity of FB-MRI	95.2%	[8]
Specificity of FB-MRI	92.0%	[8]
Costs		
Cost of XM	$ 101.52	Medicare (G0202)
Cost of DBT	$ 214.20	[23, 24]
Cost of FB-MRI	$ 314.00	Medicare (CPT code 77047)
No further action (true negative)	$ 0.00	Assumption
Biopsy	$ 1,536.00	Medicare (CPT code 19083)
Cost of treatment for tumor < 1 cm	$ 60,637	[25]
Cost of treatment for tumor > 1 cm	$ 82,121	[25]
Cost of treatment for advanced stage breast malignancy	$ 129,387	[25]
Utilities		
QOL of patients without detected tumor	1.00	Assumption
QOL of patients with detected tumor < 1 cm	0.87	[26]
QOL of patients with detected tumor > 1 cm	0.74	[27]
QOL of patients with detected regional breast cancer in an advanced stage	0.62	[28]
QOL of patients post simple treatment	0.99	Assumption
QOL of patients post intensive treatment	0.95	Assumption
Reduction in QOL due to false positive finding	0.01	Assumption
Death	0.00	Assumption
Transition probabilities		
Risk of death without tumor (yearly)	Age adjusted	US Life Tables 2017, women of all ethnicities [29]
Risk of death with undetected tumor	10.00% in 10 years	Assumption
Risk of death with detected < 1 cm tumor	0.11%	[30]
Risk of death with detected > 1 cm tumor	0.78%	[30]
Risk of death with detected tumor in advanced stage	1.81%	[30]
Probability of initial R0 resection < 1 cm	100.00%	Assumption
Probability of initial R0 resection ≥ 1 cm	90.00%	[31]
Proportion of N+ in < 1 cm tumors	0.00%	Assumption
Proportion of N+ in > 1 cm tumors	40.00%	[32]
Proportion of successfully treated tumors < 1 cm if detected within 1 screening interval	100.00%	Assumption

Estimates for the quality of life (QOL) of each state of the model were extracted from literature. Based on the size of the tumor and stage of disease, lower levels of utility were assumed for larger tumors in order to reflect therapy-related differences in quality of life [26–28]. Long-term QOL was assumed to be impaired in patients with extensive surgery and systemic treatment as opposed to QOL after treatment of small tumors. The absence of breast cancer was characterized by unimpaired utility levels.

Medicare current procedural terminology (CPT) codes and reported average costs were used to model the costs of diagnostic procedures and breast cancer therapy [23–25]. Since commonly accepted costs per examination of AB-MRI are unavailable, Medicare costs of a regular MRI examination of both breasts were applied in the base case analysis and varied in sensitivity analyses. Breast cancer incidence and tumor-unrelated as well as tumor-related death rates and the probability of R0 resection and nodal disease were extracted from literature [21, 29–32].

### Economic modelling and cost-effectiveness analysis

#### Decision model

**Fig. 1:**
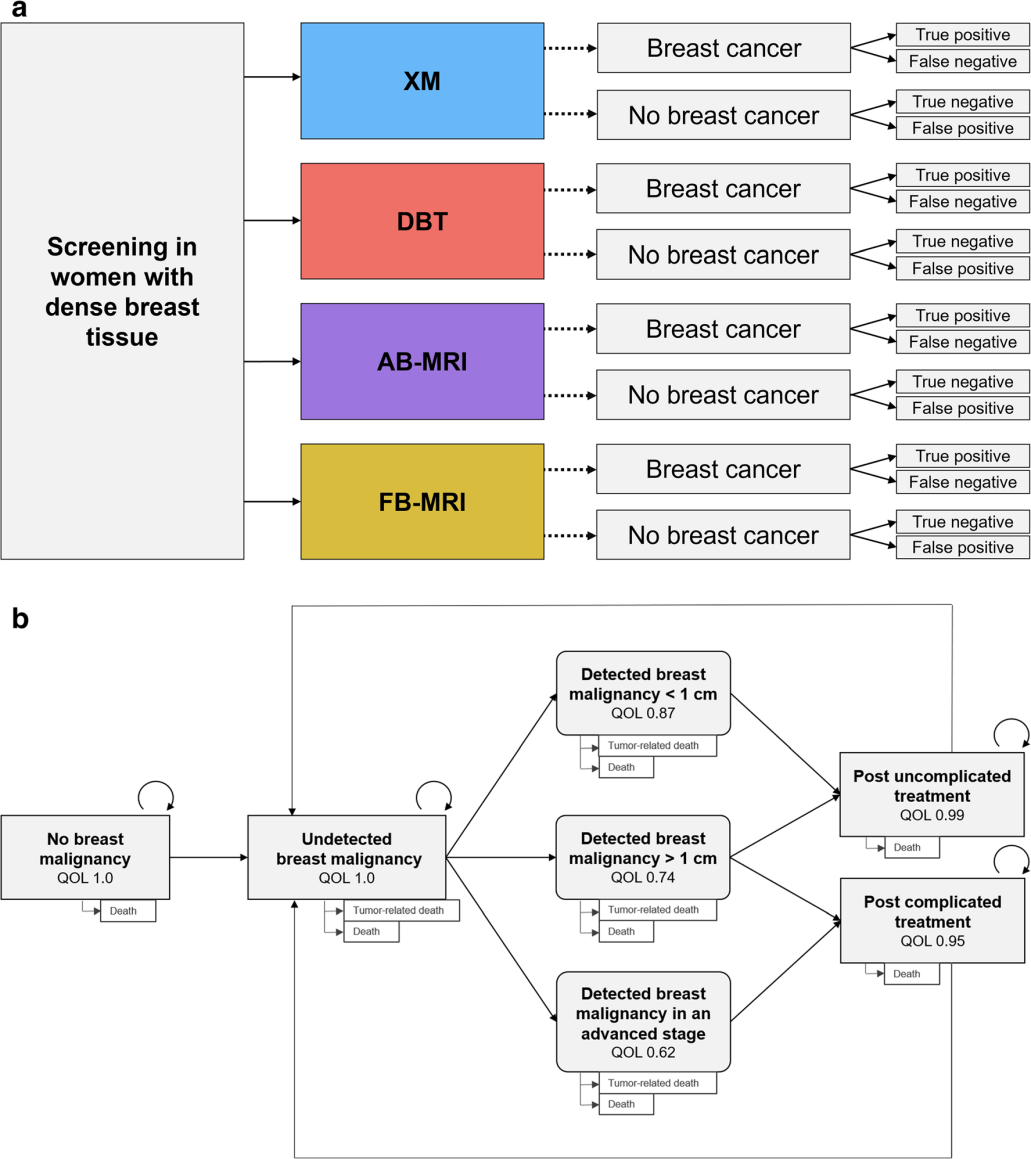
**a** Decision-tree including the four screening strategies and the corresponding outcomes true positive, false negative, true negative, and false positive. **b** Markov model with Markov states and their corresponding quality of life (QOL). The likelihood of detecting a breast tumor in two-yearly screening depends on the sensitivity of the screening method. Death is possible at any state. The Markov model has been published recently and refined for this analysis and patient collective [15, 17]. AB-MRI, abbreviated breast magnetic resonance imaging; DBT, digital breast tomosynthesis; FB-MRI, full protocol breast magnetic resonance mammography; QOL, quality of life; XM, x-ray mammography

In order to integrate results in a broader perspective and to allow for a translational comparison, we included not only MR-based screening modalities but also x-ray-based techniques. A decision model including the screening modalities was designed, and the outcomes true positive, false positive, true negative, and false negative were defined for each screening strategy (Fig. 1a). The probability of each outcome depended on the pre-test probability of a malignant lesion and the diagnostic accuracy of each modality.

#### Markov model

As described in previous publications, we adapted a Markov model for two-yearly screening programs for this study [15, 35]. Markov states included the absence of disease, undetected and detected breast cancer, long-term follow-up after initial treatment and death (Fig. 1b). False positive findings resulted in follow-up examinations and loss in quality of life. False negative findings resulted in delayed detection of breast malignancy and increased likelihood of advanced disease with more invasive and costly therapies. Positive findings in XM, DBT, and FB-MRI were histologically clarified by biopsy whereas positive findings of AB-MRI were verified or followed up by a full protocol examination and in case of a confirmed finding, resulted in biopsy. A screening interval of 2 years was assumed for the collective of women with dense breast tissue.

#### Economic analysis

The model-based cost-effectiveness analysis was conducted with a dedicated software for economic modelling and decision analysis (TreeAge Pro 2020, TreeAge Software). The perspective of the US healthcare system was chosen and costs were measured in US-$. Outcomes were modeled by using QALYs in order to consider both the length and quality of life. International recommendations for discounting costs and outcomes at an annual discount rate of 3% were applied [22]. Various willingness to pay (WTP) thresholds of $ 50,000 per QALY and $ 100,000 per QALY were assumed [36, 37]. Costs and effects were simulated for a time frame of 30 years.

### Modelling outcomes and value of AB-MRI

In the base case scenario, costs per examination of AB-MRI were assumed to equal a full protocol MRI acquisition. In subsequent deterministic sensitivity analyses, costs were reduced and the resulting cost-effectiveness was evaluated. Since the reported specificity is the main difference between abbreviated and FB-MRI, and a range of values has been reported recently, specificity was varied within plausible ranges in the sensitivity analyses.

When the cost per examination of AB-MRI is assumed smaller than the cost of FB-MRI, the incremental cost-effectiveness ratio can be calculated. When reducing the costs per examination of AB-MRI below a certain value (threshold 1), the average cumulative costs of the AB-MRI-strategy will become smaller than those of the FB-MRI-strategy. Due to its superior effectiveness, FB-MRI will still be cost-effective at a WTP threshold of $ 50,000 per QALY ($ 100,000 per QALY), until the cost per examination of AB-MRI is reduced below threshold 2 (threshold 3). AB-MRI will be the dominant strategy if costs per examination of AB-MRI are below threshold 2 or threshold 3, depending on the WTP.

In order to indicate the economic value of AB-MRI for varying costs per examination and specificity of AB-MRI, a two-way sensitivity analysis was conducted to determine the preferred modality in terms of the net monetary benefit.

## Results

### Cost-effectiveness analysis

Assuming the same cost per examination for AB-MRI as for a full protocol examination in the base-case analysis, i.e., $ 314 per examination, AB-MRI and FB-MRI resulted in average cumulative costs of $ 9,779 and $ 9,283 and outcomes of 19.25 and 19.26 QALYs, respectively. AB-MRI performed worse than FB-MRI in this simulation due to its inferior diagnostic performance, hence increased false positive findings and higher expenses for follow-up examinations.

**Fig. 2:**
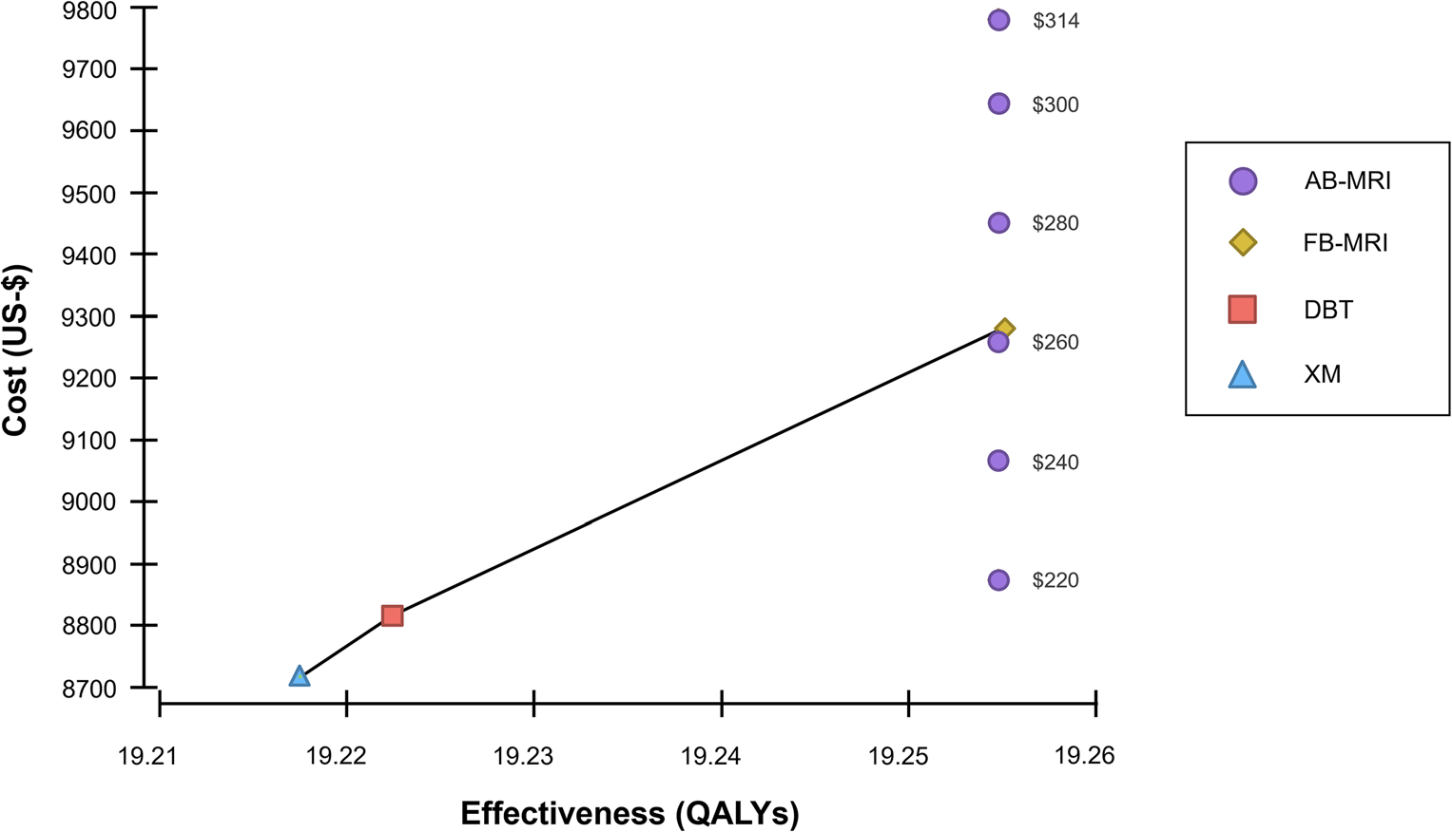
Cost-effectiveness graph for the screening strategies. Varying costs per examination of abbreviated breast MRI (AB-MRI) from US-$ 220 to US-$ 314 were assumed, which equals the cost of a full protocol breast MRI (FB-MRI). MR-based screening modalities result in higher effectiveness compared to x-ray-based modalities, but also higher average costs. AB-MRI, abbreviated breast magnetic resonance imaging; DBT, digital breast tomosynthesis; FB-MRI, full protocol breast magnetic resonance mammography; QALY, quality-adjusted life year; XM, x-ray mammography

To put the results in a broader perspective, we respectively included cumulative costs and outcomes for conventional imaging as well: cumulative costs and effects over a time horizon of 30 years were larger for MR-based diagnostic procedures than for x-ray-based procedures in the base case analysis (Table 2 and Fig. 2).

**Table 2:** Results of cost-effectiveness analysis. Results of the base-case cost-effectiveness analysis and of variations of the price per examination of AB-MRI. All values reference a common baseline (XM)

Strategy (cost per examination in US-$)	Cumulative discounted costs (US-$)	Incremental costs (US-$)	Cumulative discounted effects (QALYs)	Incremental effects (QALYs)	ICER (US-$/QALY)
XM $ 102	8,718	n/a	19.22	n/a	n/a
DBT $ 214	8,815	97	19.22	0.005	19,785
AB-MRI					
$ 314	9,779	1,062	19.25	0.037	28,458
$ 300	9,644	927	19.25	0.037	24,840
$ 280	9,452	734	19.25	0.037	19,671
$ 260	9,259	541	19.25	0.037	14,501
$ 240	9,066	348	19.25	0.037	9,332
$ 220	8,873	155	19.25	0.037	4,163
$ 200	8,680	-38	19.25	0.037	n/a
FB-MRI $ 314	9,283	565	19.26	0.038	15,018

*ICER*, incremental cost-effectiveness analysis; *QALY*, quality-adjusted life year; *XM*, x-ray mammography; *DBT*, digital breast tomosynthesis; *AB-MRI*, abbreviated breast magnetic resonance imaging; *FB-MRI*, full protocol breast magnetic resonance mammography

### Abbreviating breast MRI

In the next step, the assumed cost per examination of AB-MRI was reduced and the model outputs and the resulting cost-effectiveness were evaluated accordingly.

**Fig. 3:**
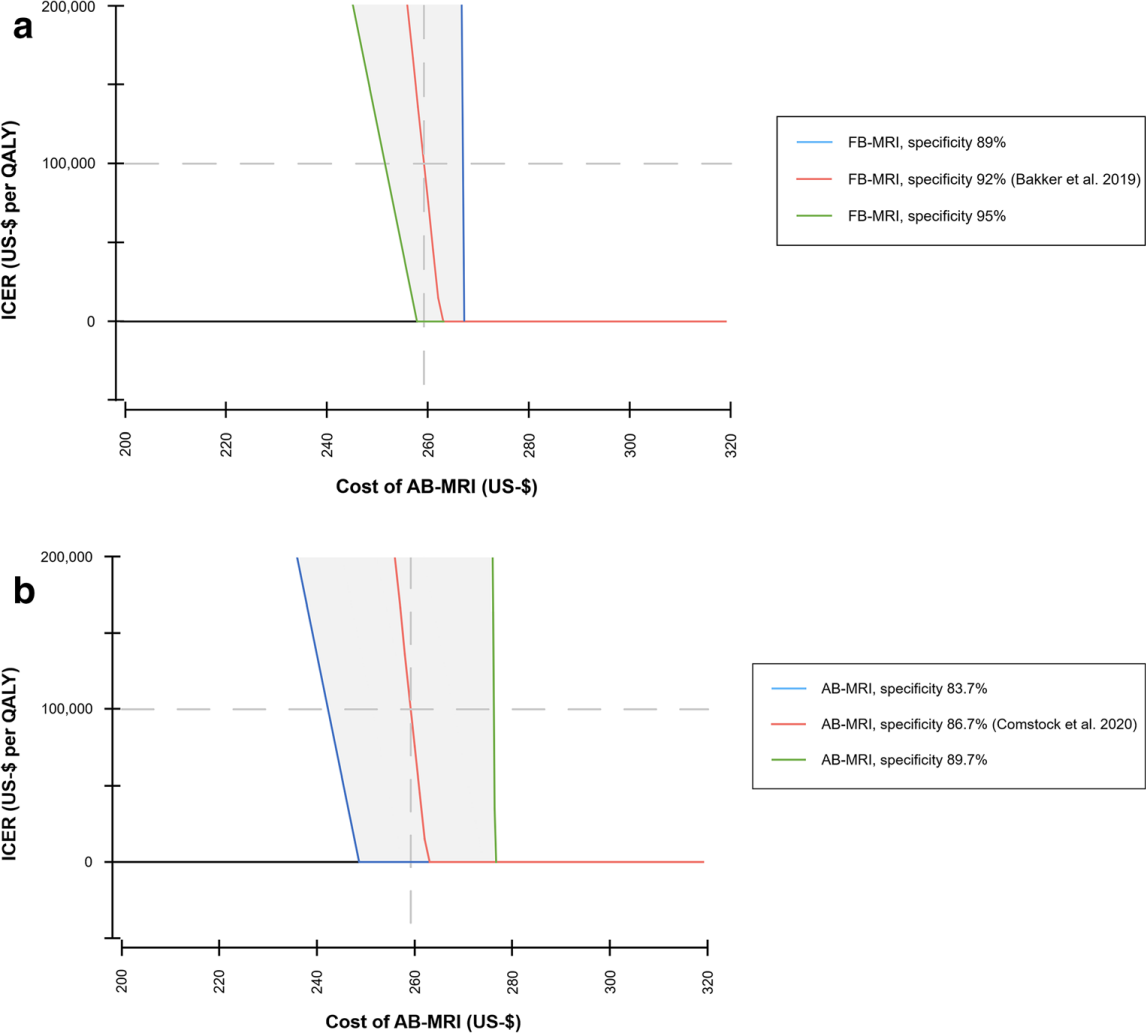
One-way sensitivity analysis for varying costs per examination of abbreviated breast MRI (AB-MRI). The resulting incremental cost-effectiveness ratio (ICER) of full protocol breast MRI (FB-MRI) compared to AB-MRI is indicated for varying specificities of FB-MRI (**a**) and varying specificities of AB-MRI (**b**). For a specificity of 92%, FB-MRI is cost-saving if the cost per examination of AB-MRI is larger than $ 263 (threshold 1). The ICER of FB-MRI is below a willingness to pay threshold of $100,000 per QALY if the cost per examination of AB-MRI is between $ 259 and $ 263 (threshold 2), indicating AB-MRI to be cost-effective below $ 259. AB-MRI, abbreviated breast magnetic resonance imaging; FB-MRI, full protocol breast magnetic resonance mammography; ICER, incremental cost-effectiveness ratio; QALY, quality-adjusted life year

**Fig. 4:**
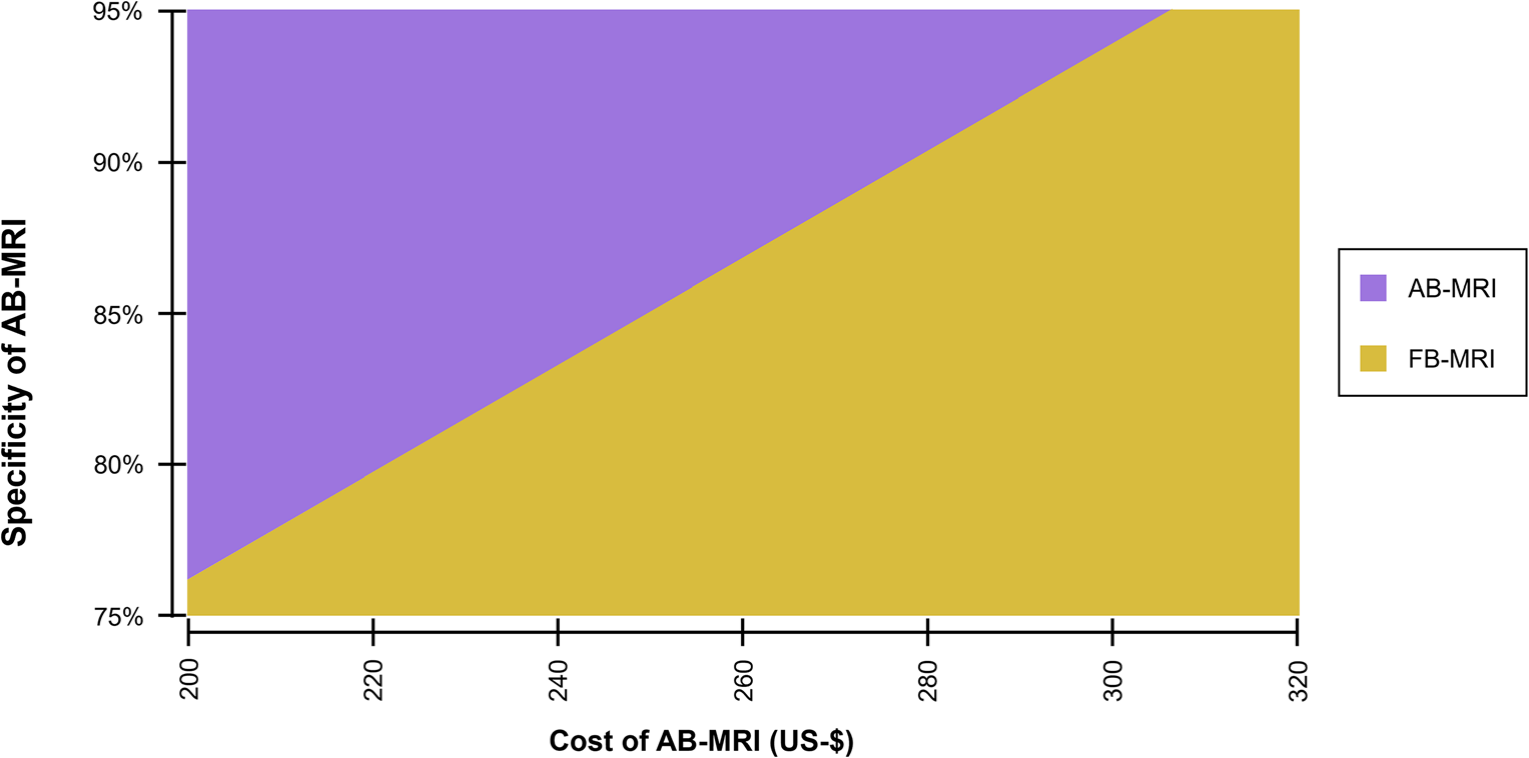
Two-way sensitivity analysis of the net monetary benefit for varying cost per examination and varying specificity of abbreviated breast MRI (AB-MRI). The color-coded area indicates the dominant strategy at a given cost and specificity of AB-MRI. The smaller the cost and the higher the specificity of AB-MRI, the more likely it ought to be preferred to full protocol breast MRI (FB-MRI). AB-MRI, abbreviated breast magnetic resonance imaging; FB-MRI, full protocol breast magnetic resonance mammography

Reducing the cost per examination of AB-MRI resulted in decreased discounted cumulative costs over the time horizon of 30 years and improved cost-effectiveness in terms of ICER and net monetary benefits (Table 2, Fig. 3 and Fig. 4).

At a cost per examination of $ 263 for AB-MRI (84% of the MEDICARE cost of a FB-MRI examination), the average cumulative costs of both MR-based strategies were comparable (Fig. 3). When the cost per examination of $ 259 was assumed for AB-MRI (82% of the cost of a FB-MRI examination), the average cumulative costs decreased to $ 9,249. Comparing FB-MRI to AB-MRI in a collective of 1,000 women, this resulted in incremental costs of $ 31,827 and incremental effects of 0.32 QALYs for FB-MRI, which is reflected by an ICER of $ 100,000 per QALY (WTP threshold). Reducing the cost of AB-MRI below $ 259, the average cumulative costs over the time horizon of 30 years were reduced even further, so that the ICER of FB-MRI compared to AB-MRI exceeded the WTP threshold of $ 100,000 per QALY and the AB-MRI strategy ought to be preferred. This means that the cost per examination of AB-MRI would be required not to exceed $ 259 (82% of the cost of a FB-MRI examination), in order to stay cost-effective. Any cost above this threshold would deem FB-MRI a feasible and cost-effective alternative.

### Sensitivity analysis

Abbreviating breast MRI affects the performance of the technique mainly in terms of specificity, i.e., its reduced specificity and a higher number of false positive findings. To evaluate the impact of varying specificities on the model outputs, sensitivity analyses were conducted. The cost cut-off at which the AB-MRI strategy may be considered cost equal to the FB-MRI strategy (threshold 1) and the threshold at which AB-MRI should be preferred over FB-MRI (threshold 2) were simulated (Table 3). For example, at a specificity of 95% for FB-MRI and 87% for AB-MRI, the cumulative costs of both strategies were equal when the cost per examination of AB-MRI was set to $ 260 (83% of the cost of a FB-MRI examination). Below costs per examination of $ 253 (81%), FB-MRI was no longer a cost-effective alternative based on a WTP threshold of $ 100,000 per QALY. This emphasizes the need for AB-MRI to not exceed $ 253 in order to stay cost-effective in this scenario.

**Table 3:** Cost threshold analysis. Cost threshold analysis for varying specificity of AB-MRI and full protocol breast MRI (FB-MRI). If the cost per examination of AB-MRI was higher than threshold 1, the FB-MRI-strategy would be cost-saving. If the cost per examination of AB-MRI was higher than threshold 2 or threshold 3, FB-MRI would be cost-effective at a willingness-to-pay threshold of $ 50,000 per QALY or $ 100,000 per QALY, respectively. If the cost per examination of AB-MRI was below the second or third threshold-value, AB-MRI should be preferred since FB-MRI would not be cost-effective. The cost thresholds are reported in absolute values in US-$ and in relation to the cost of a FB-MRI examination, which was defined at $ 314 according to Medicare CPT codes

Specificity of AB-MRI	Specificity of FB-MRI				
	83%	86%	89%	92%	95%
81%	$ 249 (79%), $ 249 (79%), $ 249 (79%)	$ 246 (78%), $ 244 (78%), $ 242 (77%)	$ 241 (77%), $ 238 (76%), $ 235 (75%)	$ 237 (75%), $ 231 (74%), $ 227 (72%)	$ 232 (74%), $ 225 (72%), $ 219 (70%)
83%	-	$ 255 (81%), $ 254 (81%), $ 254 (81%)	$ 250 (80%), $ 248 (79%), $ 246 (78%)	$ 246 (78%), $ 242 (77%), $ 238 (76%)	$ 241(77%), $ 236 (75%), $ 231 (74%)
85%	-	$ 264 (84%), $ 264 (84%), $ 265 (84%)	$ 260 (83%), $ 258 (82%), $ 257 (82%)	$ 255 (81%), $ 252 (80%), $ 250 (80%)	$ 250 (80%), $ 246 (78%), $ 242 (77%)
87%	-	-	$ 268 (85%), $ 268 (85%), $ 268 (85%)	$ 265 (84%), $ 262 (83%), $ 261 (83%)	$ 260 (83%), $ 256 (82%), $ 253 (81%)
89%	-	-	-	$ 273 (87%), $ 273 (87%), $ 272 (87%)	$ 270 (86%), $ 266 (85%), $ 264 (84%)

Figure 4 indicates which strategy was to be preferred in terms of net monetary benefit when cost per examination of AB-MRI and specificity of AB-MRI were varied.

## Discussion

The superior diagnostic performance of breast MRI in screening women with dense breast tissue for breast cancer in comparison to x-ray-based techniques has been investigated and confirmed in recent prospective multi-center studies [7, 8].

A significant reduction in interval cancer rates as an accepted surrogate marker for mortality [38] was observed in the DENSE trial when screening women with elevated breast tissue densities with breast MRI compared to XM [8]. A recent publication demonstrated that additionally to its diagnostic benefit, FB-MRI may also be considered cost-effective in this setting, evaluating long-term costs and outcomes of screening women at intermediate risk of breast cancer due to their elevated breast density [15].

AB-MRI was introduced in 2014 in order to reduce both examination as well as image reading time, i.e., the costs per examination, while maintaining an acceptable diagnostic performance for reasons of cost-effectiveness [10]. The initial concept by Kuhl et al comprised only one pre- and one post-contrast acquisition with computation of a first post-contrast subtracted image and a maximum-intensity projection-image. In the following years, various protocols with and without contrast agent, some only based on diffusion-weighted imaging or ultrafast contrast-enhanced sequences, have been proposed in order to make breast MRI supposedly less expensive and thereby more cost-effective [11–14]. However, the reduction in examination costs at the expense of reduced specificity must economically be considered a trade-off.

Recently, Comstock et al demonstrated a significantly higher rate of breast cancer detection for AB-MRI compared to DBT [7]. Instead of applying only *one* method of abbreviation, Comstock et al accepted various abbreviated protocols in their multi-center study with 48 participating imaging centers, with pre- and post-contrast sequences and T2-weighted imaging as long as a total examination time of 10 minutes was not exceeded. A recent publication demonstrated that AB-MRI may be cost-effective compared to DBT in screening women with dense breast tissue [17].

From an economical perspective, however, the degree of abbreviation and the resulting cost-effectiveness of the different forms of abbreviating protocols is of major interest when extending MR-based breast cancer screening to a population level. This led to the main goal of the present study as to determine the optimal balance of price vs. specificity of AB-MRI — assuming a standard Medicare reimbursement for breast MRI of $ 314.

A reduction of imaging specificity resulted in an increase of false positive findings and follow-up scan rates as well as a reduction in quality of life of the affected women. These downsides need to be put in relation to these cost reductions achieved by AB-MRI. However, AB-MRI has only rarely been investigated within clinical trials investigating intermediate-risk collectives. Up to this day, there is no broadly accepted definition as to the effective reduction of costs by applying different sets of abbreviated protocols. We therefore varied the costs per examination of AB-MRI in our study within our sensitivity analyses.

Comparing the specificities for breast MRI in a screening setting for women with dense breast tissue, we were able to determine a cut-off value of approximately 80% of the Medicare reimbursement of a FB-MRI examination ($ 259).

In other words: Assuming a reduced specificity of AB-MRI in an intermediate-risk population, the cost of AB-MRI should not exceed $ 259 in order to be preferred in terms of cost-effectiveness. This analysis may be considered the first step towards an understanding of the economic potential of AB-MRI on a population-based level.

However, some limitations of the model-based economic evaluation need to be considered:

The selected WTP thresholds of $ 50,000–$ 100,000 have been subject to scientific debate, yet have been applied to the U.S healthcare system by the majority of economic evaluations and represent the commonly accepted standard.

A Markov model can only represent an approximation of clinical reality and is characterized by its input parameters. The values of the diagnostic performance are based on two randomized prospective multi-center studies that were conducted in slightly different populations with different baseline risks: the DENSE trial included women with breast density category D, whereas Comstock et al included women with categories C and D. Breast tissue density hereby represents certainly only one risk factor among others that can contribute to an intermediate risk profile.

Importantly, sensitivity and specificity of breast MRI are independent from mammographic breast tissue density [39]. Therefore, the absolute values of diagnostic performance reported by Comstock and Bakker could reasonably be applied to this study population. An average pre-test probability of malignancy as reported by Comstock and Bakker and an average incidence rate were included into the economic model for comparing both screening strategies in order to deal with the heterogeneity of the two underlying studies. Further, the study design was independent from breast density as diagnostic performances of both FB-MRI and AB-MRI are unaffected by breast tissue density; the goal was merely to compare different specificity levels of the underlying methods of examination. Comparing different risk-stratified screening strategies across various screening populations with different grades of breast tissue density was not an aim of this analysis. The comparison of MR-based techniques with XM and DBT was not feasible since performance measures of x-ray-based techniques were acquired from different populations with divergent breast tissue densities.

In the DENSE concept biennial screening with conventional mammography was supplemented with breast MRI. Comstock et al on the contrary applied annual screening. Evidence-based recommendations on the selection of appropriate screening intervals for screening women with dense breasts with MRI are so far unavailable. We therefore selected a biennial screening interval for our economic evaluation, until prospective data comparing annual with biennial in terms of diagnostic performance and effects on mortality are scientifically explored further. Recent literature has suggested the potential economic value of a 4-year screening interval [40]. However, we did not include overly distant screening intervals of 4 years or more into our analysis since empirical outcome data on the interval cancer rates and stages of screening-detected breast cancer are unavailable as of today.

Additionally, both studies only describe the results of only the first screening round. However, recent findings of the second screening round of the DENSE trial indicate a decrease of false positives when breast MRI is routinely applied as a screening tool [41]. Even though the initial definition of protocol abbreviation as described by Kuhl et al suggested a FB-MRI examination after an initially suspicious finding, abbreviated protocols have not been clearly defined or standardized so far. Comstock et al included varying protocols as defined above and did not consider appending a FB-MRI examination in case of unclear findings.

Just as FB-MRI is merely defined by its fixed reimbursement and not ultimately by the composition of its protocol, further research as to the optimal degree of abbreviation and set of sequences is needed.

Further studies are required in order to analyze the role of other sources of varying specificity, such as reader experience.

At the same time, technical advances, e.g., by parallel imaging, allow for shorter examination times without possible detriments to diagnostic performance [42]. Hence, the gap between abbreviated protocols and full protocols may diminish to some extent in the future. Based on these developments, we deem recommendations on the role of AB-MRI in screening women with dense breasts premature as of today.

In conclusion, our preliminary findings offer a first delineation of the economic value of AB-MRI compared to FB-MRI and indicate certain cost thresholds that should not be exceeded in order to maintain a preferable cost-effectiveness of AB-MRI over FB-MRI.

## Abbreviations

AB-MRI: Abbreviated breast MRI
DBT: Tomosynthesis
FB-MRI: Full protocol breast MRI
ICER: Incremental cost-effectiveness ratio
MRI: Magnetic resonance imaging
PSA: Probabilistic sensitivity analysis
QALY: Quality-adjusted life year
QOL: Quality of life
WTP: Willingness to pay

## Declarations

### Guarantor

The scientific guarantor of this publication is CG Kaiser.

### Statistics and biometry

Several of the authors have significant statistical expertise.

### Methodology

• not applicable

• not applicable

• performed at one institution
